# Enhancement of bradykinin-induced relaxation by focal brain ischemia in the rat middle cerebral artery: Receptor expression upregulation and activation of multiple pathways

**DOI:** 10.1371/journal.pone.0198553

**Published:** 2018-06-18

**Authors:** Youhai Li, Natalia Lapina, Nina Weinzierl, Lothar Schilling

**Affiliations:** Division of Neurosurgical Research, Medical Faculty Mannheim, Heidelberg University, Mannheim, Germany; Texas Tech University, UNITED STATES

## Abstract

Focal brain ischemia markedly affects cerebrovascular reactivity. So far, these changes have mainly been related to alterations in the level of smooth muscle cell function while alterations of the endothelial lining have not yet been studied in detail. We have, therefore, investigated the effects of ischemia/reperfusion injury on bradykinin (BK)-induced relaxation since BK is an important mediator of tissue inflammation and affects vascular function in an endothelium-dependent manner. Focal brain ischemia was induced in rats by endovascular filament occlusion (2h) of the middle cerebral artery (MCA). After 22h reperfusion, both MCAs were harvested and the response to BK studied in organ bath experiments. Expression of the BK receptor subtypes 1 and 2 (B_1_, B_2_) was determined by real-time semi-quantitative RT-qPCR methodology, and whole mount immunofluorescence staining was performed to show the B_2_ receptor protein expression. In control animals, BK did not induce significant vasomotor effects despite a functionally intact endothelium and robust expression of B_2_ mRNA. After ischemia/reperfusion injury, BK induced a concentration-related sustained relaxation in all arteries studied, more pronounced in the ipsilateral than in the contralateral MCA. The B_2_ mRNA was significantly upregulated and the B_1_ mRNA displayed *de novo* expression, again more pronounced ipsi- than contralaterally. Endothelial cells displaying B_2_ receptor immunofluorescence were observed scattered or clustered in previously occluded MCAs. Relaxation to BK was mediated by B_2_ receptor activation, abolished after endothelium denudation, and largely diminished by blocking nitric oxide (NO) release or soluble guanylyl cyclase activity. Relaxation to BK was partially inhibited by charybdotoxin (ChTx), but not apamin or iberiotoxin suggesting activation of an endothelium-dependent hyperpolarization pathway. When the NO-cGMP pathway was blocked, BK induced a transient relaxation which was suppressed by ChTx. After ischemia/reperfusion injury BK elicits endothelium-dependent relaxation which was not detectable in control MCAs. This gain of function is mediated by B_2_ receptor activation and involves the release of NO and activation of an endothelium-dependent hyperpolarization. It goes along with increased B_2_ mRNA and protein expression, leaving the functional role of the *de novo* B_1_ receptor expression still open.

## Introduction

The presence of kinins, as well as kinin-synthesizing and–destroying enzymes in the brain, was first described by Hori [1] in rabbits. Based on these and subsequent studies supporting and extending the initial findings, brain tissue is considered to express a full kallikrein-kinin system (KKS). The major biologically active component of the KKS, the nonapeptide bradykinin (BK), acts upon two receptor types termed subtype 1 (B_1_) and subtype 2 (B_2_) receptor. In the brain vasculature, BK exerts endothelium-dependent vasodilatation of arteries and arterioles and an increase in capillary permeability eventually leading to (enhancement of) vasogenic brain edema. In healthy conditions, these effects are mediated by activating the B_2_ receptor [2] which is constitutively expressed on the endothelial cells while the expression of the B_1_ receptor is typically below detection levels.

Despite much research performed in the past, the importance of the B_1_ and B_2_ receptors in vascular dysfunction and tissue damage following a traumatic or ischemic brain insult is still not fully understood. A major role of the B_2_ receptor may be derived from studies using selective receptor antagonists or B_2_ receptor knock-out mice showing a reduction of the volume of tissue, a decrease of brain edema, and improvement of neurological outcome in models of brain trauma [3–6] and ischemia [7–9]. Moreover, in brain trauma patients, application of B_2_ receptor antagonists proved effective in counteracting the increase in intracranial pressure [10, 11]. However, pharmacological inhibition or knock-out of the B_1_ receptor has also been shown to carry therapeutic benefit in a model of ischemic brain damage in mice [12]. Thus, both B_1,_ as well as B_2_ receptors, may be of functional importance in KKS activation and mediation of BK-induced effects in pathological situations.

Alterations of cerebroarterial reactivity in pathological conditions accompanied by alterations of gene expression have been demonstrated for endothelin-1, angiotensin II, and 5-HT in models of focal brain ischemia [13–15]. However, these studies have focused on the smooth muscle cells only, disregarding potential changes in endothelial cells, which play a pivotal role in the regulation of vascular tone and reactivity. We have, therefore, investigated the alterations of the vasomotor effects of BK following ischemia/reperfusion (I/R) injury in the rat middle cerebral artery (MCA). Bradykinin was chosen because (i) its vasomotor effect in cerebral arteries is endothelium-dependent, and (ii) activation of the KKS is a highly adaptive inflammatory response following ischemic and traumatic injury with a *de novo* expression of the B_1_ receptor appearing to be a hallmark [16]. Although B_1_ receptor activation has been linked to vasomotor activity in peripheral arteries [17], no such data are available yet in cerebral arteries.

Our results indicate a tremendous increase in the relaxation-inducing action of BK in rat MCA following I/R injury. Despite a *de novo* expression of the B_1_ receptor mRNA in the vessel wall, we found no indication of any vasomotor effect. In fact, relaxation mediated by B_2_ receptor activation was endothelium-dependent and involved release of nitric oxide (NO) and activation of an endothelium-dependent hyperpolarizing factor (EDHF)-related pathway. The enhanced vasomotor action went along with a significant upregulation of the B_2_ receptor on the level of gene expression and occurrence of a respective immunofluorescent signal in the endothelium. To the best of our knowledge, the present study is the first to describe I/R injury-related alterations of B_1_ and B_2_ receptors on the levels of gene expression, protein expression and vasomotor reactivity in the rat MCA.

## Materials and methods

Male Sprague Dawley rats (body weight 300–500 g) from Janvier (Isle St. Genest, France) were used throughout. All animals were allowed to accommodate for at least one week before experiments were done. Approval of the experimental protocol was obtained from the Federal Animal Ethics Committee (Regierungspraesidium Karlsruhe). The experiments were performed in compliance with the relevant laws and institutional guidelines for the care and use of animals in research according to the Directive 2010/63/EU. This includes all efforts to minimize pain and stress to the animals and limits to the number of animals used.

### Induction of focal ischemia and reperfusion

Focal brain ischemia was induced by the intravascular filament occlusion technique as described previously [18, 19]. A surgical level of anesthesia was established with isoflurane (3.0% in a 70:30 air: O_2_ mixture) delivered through a face mask while spontaneously breathing. The skull was exposed and a hole created over the right (subsequent ischemic) hemisphere (1mm caudally / 4 mm laterally from the bregma) leaving the inner bone layer intact. A glass fiber connected to a laser Doppler flowmetry (LDF) monitor (Moore DRT 4, Axminster, England) was attached to the hole for continuous recording of perfusion in the area supplied by the MCA. The animal was then turned into a supine position and the neck opened in the midline. The carotid bifurcation was identified on the right side and flow through the common carotid artery transiently blocked. The external carotid artery was ligated and mobilized. A nylon filament (4–0) coated with an elastomer (Provil novolight, Heraeus Kulzer, Hanau Germany; tip diameter, 430–460 μm) was introduced and advanced into the internal carotid artery until a sudden drop of the LDF signal indicated MCA occlusion. The filament was fixed to the external carotid artery, the block of the common carotid artery released and the LDF recording checked for signs of hemorrhage [20]. Buprenorphine (3 μg / 100 g body weight) was injected subcutaneously to reduce postoperative pain, the glass fiber was removed, and after closure of all wounds, the animal was returned to its cage to recover from anesthesia.

The animals were re-anesthetized with isoflurane 100 min after establishing MCA occlusion. The LDF-probe was re-inserted in the skull and the filament removed 2 h after positioning. A sharp increase of the LDF signal indicated successful reperfusion. Buprenorphine was again injected (half the dose given in the first surgical session) and after removal of the LDF fiber all wounds were closed again. Anesthesia was discontinued and the animal returned to its cage.

### Studies of vasoreactivity

At the end of the 24h observation period, the animals were deeply anesthetized (5% isoflurane in oxygen) and sacrificed by bleeding from the carotid arteries. The skull was quickly opened and the brain carefully removed and transferred into a dish containing cold modified Krebs solution (composition in mM: NaCl, 119; KCl, 3.0; NaH_2_PO_4_, 1.2; CaCl_2_, 1.5; MgCl_2_, 1.2; NaHCO_3_, 15; glucose, 10). Both MCAs were meticulously isolated under a binocular microscope (GZ6, Zeiss, Oberkochem, Germany) and ring segments of approximately 2 mm length mounted onto 2 stainless steel wires (diameter, 30μm) for measurement of isometric force. In some experiments endothelium denudation was performed by rubbing the luminal surface of the segment with an eyelash as described previously [21].

After mounting, all segments received a 90 min accommodation period with warming of the bath solution to 37°C and repeated correction of the resting tension until a stable level of approximately 2.5 mN was reached. Tension was measured by force transducers (F10, coupled to TAM-A amplifiers, both from Hugo Sachs, March-Hugstetten, Germany). The signals from the force detection systems were fed into a computer and continuously recorded using a purpose-built program based on LabView (Munich, Germany) as described previously [22].

After accommodation all segments underwent standard tests to prove (i) the functional integrity of the contractile apparatus by immersion in Krebs solutions containing increasing concentrations of K^+^ (50, 80, and 124 mM; NaCl substituted by KCl in equimolar amounts) and (ii) the presence or successful removal of the endothelium by cumulative application of sarafotoxin 6c (10^−8^ and 10^−7^ M) following precontraction with the thromboxane A_2_ receptor analogue U46619 (3x10^-7^ M). Sarafotoxin 6c selectively activates the endothelin B receptor subtype located in endothelial cells resulting in release of NO [23].

The vasomotor effect of BK was studied after precontraction with U46619 (we never observed contraction when BK was applied upon resting tension). Receptor characterization was done by incubating ring segments for at least 30 minutes with the selective B_1_ receptor antagonist lys-(des-arg^9^,leu^8^)-BK (10^−5^ M) or the selective B_2_ receptor antagonist Hoe140 (10 ^-6^ M) before application of BK. In addition, we also tested the effect of the selective B_1_ receptor agonist lys-(des-arg^9^)-BK (10^−12^–10^−6^ M) in precontracted segments. In order to characterize the mechanisms involved in mediating BK-induced relaxation after I/R injury, we used blockers of NO release and inhibitors of Ca^2+^-dependent K^+^ (K_Ca_) channels. In each case, the segments were exposed to the blockers for at least 30 minutes before precontraction with U46619 and application of BK.

### Studies of gene expression

Gene expression was studied in individual MCAs. The arteries were carefully cleaned from all adhering tissue, transferred into cell lysis buffer (RLT buffer [Qiagen, Hilden, Germany], 350 μl plus 3.5 μl mercaptoethanol), and immediately cooled down to -75°C until further use. Each MCA underwent mechanical disruption using a motor-driven homogenizer (Homgen, Schuett Biotec, Goettingen, Germany), shearing of DNA by repeated pushing through a 30G needle, and extraction of total RNA using a commercially available kit (RNeasyplus, Qiagen). Concentration and integrity of the total RNA obtained were measured using Picochips read in a 2100 Bioanalyzer (both from Agilent Technologies, Waldbronn, Germany).

For reverse transcription 10μl total RNA solution obtained from individual arteries was mixed with 1 μl random hexamers (200 ng/μl) and 1 μl oligo (dT)_18_ solution (10 μM; both from Sigma-Aldrich), heated to 70° C for 5 min and immediately cooled down on ice. Thereafter, 8 μl master mix (4 μl 5x RT-buffer; 2 μl dNTP mix [5 mM each], 1 μl RNase Inhibitor [RNasin® Plus, 20 U/μl]; all from Promega), 1 μl Sensiscript^®^ reverse transcriptase [200 U/μl]; Qiagen) were added and the solution was incubated at 37° C for 90 min (PCR Express, Hybaid Thermo Scientific). The reaction was stopped by increasing to 94° C for 10 min.

Gene expression was measured by real-time semi-quantitative real-time PCR (RT-qPCR) methodology using 1 μl of cDNA solution which was mixed with 8 μl PCR-grade H_2_O, 10 μl Absolute™QPCR Mix (Thermo Scientific, Hamburg, Germany), and 1 μl of a probe specific primer mixture (TaqMan® Gene expression Assay [20x], Applied Biosystems). We used commercially available hydrolysis probes (TaqMan®, Applied Biosystems, Darmstadt, Germany). The assay designations (in brackets are given the gene name, the accession number, the respective assay number, the assay location, and the amplicon length) are elongation factor 1 (EeF1a2, NM_012660.2, #Rn00561973_m1, 1592, 82bp,), B_1_ receptor (Bdkrb1, NM_030851.1, #Rn00578261_m1, 86; 105 bp), and B_2_ receptor (Bdkrb2, NM_173100.2, #Rn00597384_m1, 209, 75bp,). The amplification protocol consisted of an initial heating period (95°C for 15 min) followed by 40 amplification cycles (95°C for 15 sec and 60°C for 1 min). Experiments were performed using either a Stratagene MxPro 3005P light cycler (Stratagene Europe, Amsterdam, The Netherlands) or a StepOne light cycler (Applied Biosystems). In preliminary studies we confirmed that both systems yielded fully comparable results. Measurements were done at least in duplicates and the cycle threshold (C_T_) values averaged to be used for further analyses.

### Immunofluorescence detection of B_2_ receptor protein expression

Whole mount immunofluorescence microscopy was performed to detect B_2_ receptor protein expression in the MCA wall. Arteries were cut along their long axis and immersed in 4% paraformaldehyde for 10 minutes at room temperature. Subsequently, free-floating staining was performed in 96 well plates at room temperature unless otherwise stated. The protocol with washes between the incubation steps consisted of (i) incubation in TritonX100 (1% dissolved in saline solution PBS for 30 minutes), (ii) immersion in serum free protein block (DAKO X0909, DAKO, Hamburg, Germany) for 60 minutes, (iii) incubation with a primary antibody raised against the B_2_ receptor protein (mouse monoclonal α B2R, sc-136216, Santa Cruz, Heidelberg, Germany; dilution, 1:300 overnight at 4°C), (iv) incubation with the secondary antiserum (goat anti mouse IgG, Invitrogen A11004 Alexa Fluor 568, Thermo Fisher Scientific, Darmstadt, Germany; dilution 1:1000 for 3 h), (v) nuclear counterstaining with 4′,6-Diamidin-2-phenylindol (DAPI, 300 nM), (vi) covering (Roti^®^-Mount FluorCare HP19.1, Carl Roth, Karlsruhe, Germany). The segments were examined under a fluorescence microscope (Z1) equipped with an Axiocam camera (both from Carl Zeiss, Jena, Germany).

### Measurement of ischemic brain damage

The volume of ischemic damage was determined based on the high contrast silver nitrate staining method introduced by Vogel and coworkers [24]. Briefly, serial cryosections of the brain (thickness, 20 μm; distance 1 mm) were air-dried, immersed in an impregnation solution for 2 min (composition: 5ml of a 10% silver nitrate solution and 10ml saturated lithium carbonate solution were mixed and after dissolving the white precipitate by adding 25% ammonia drop by drop, 75ml distilled water was added), washed in distilled water (6 x 1 min) and subsequently immersed in a developing solution for 3 min (0.3 g hydroquinone, 15 ml acetone and 1.1g trisodium citrate dissolved in 20ml formaldehyde (37%) / 70ml distilled water), washed again before covering with Eukitt medium (O. Kindler GmbH, Bobingen, Germany). Volumetric analysis of the brain damage was performed as described in detail previously [19].

### Materials

The chemicals used in the present study along with the suppliers are Bk acetate salt, lys-(des-arg^9^)-BK trifluoroacetate salt (B_1_ receptor agonist), lys-(des-arg^9^,leu^8^)-BK (B_1_ receptor antagonist) and iberiotoxin from Bachem (Heidelberg, Germany), Hoe 140 (Arg-Arg-Pro-Hyp-Gly-Thi-Ser-Tic-Oic-Arg, B_2_ receptor antagonist), charybdotoxin (ChTx), N^ω^–nitro-l-arginine (L-NNA), trisodium citrate dihydrate, hydroquinone, and carbolithium from Sigma-Aldrich (Taufkirchen, Germany); sarafotoxin 6c, U46619 (9,11-Dideoxy-9a,11a-methanoepoxy prostaglandin F_2a_) and ODQ (1H-[1,2,4]Oxadiazolo[4,3-a]quinoxalin-1-one) from Enzo Life Science (Lausanne, Switzerland); 7-nitroindazole (7-NI) sodium salt from Santa Cruz Biotechnology (Heidelberg, Germany); apamin from RBI (Köln, Germany); NaCl, KCl, MgCl_2_, NaHCO_3_, glucose, and 25% ammonia solution from ROTH (Karlsruhe, Germany); NaH_2_PO_4_, CaCl_2_, silver nitrate, formaldehyde and acetone from Merck (Darmstadt, Germany); buprenorphine hydrochloride (Temgesic™; Essex Pharma, Munich, Germany); isoflurane (Forene™, AbbVie, Ludwigshafen, Germany).

### Calculations and statistical analyses

In myograph studies relaxation is expressed in percent of precontraction induced by U46619 (300 nM). Levels of gene expression in the MCA wall were determined by means of the ΔC_T_ method with elongation factor-1 serving as house-keeping gene. Ischemia-induced alterations of gene expression were determined using the comparative threshold cycle method (ΔΔC_T_ methodology), and accumulation or depletion was calculated as fold change = 2^-ΔΔC^_T_ according to Schmittgen and Livak [25].

The SigmaPlot software package (vers. 12.5; Systat Software GmbH, Erkrath, Germany) was employed for statistical analysis and preparation of the graphs. Statistical analysis was done using unpaired Student’s t-test or if appropriate ANOVA procedure along with the Tukey test for post-hoc pairwise comparisons. All values are given as mean ±SEM along with the numbers of observations. In addition, fold changes are given along with the 95% confidence intervals.

## Results

A total of 42 rats underwent MCA occlusion. Among these 5 were excluded because of a premature death. Occlusion of the MCA origin resulted in development of focal ischemic damage throughout all animals included in the analysis. Hemispheric swelling was 36±2.5% indicating the presence of marked vasogenic edema. The volume of ischemic damage was 340±16 mm^3^ (n = 28) after appropriate correction for brain swelling.

### Studies in MCAs from control animals

In cerebral arteries obtained from control rats, BK did not induce any significant vasomotor effect, neither under resting tension conditions nor following precontraction with U46619 (3x10^-7^ M). However, in each of these segments sarafotoxin 6c induced significant relaxation (maximum effect, 57±5.2%; n = 25) indicating that the failure of BK to elicit relaxation could not be ascribed to (functional) damage of the endothelium. Despite the lack of a significant vasomotor effect we found the B_2_ message present in all MCA vessels studied by means of RT-qPCR methodology (see Table 1).

**Table 1:** Expression levels of the bradykinin subtype 1receptor (B_1_) and subtype 2 receptor (B_2_) mRNA in rat middle cerebral arteries (MCA) obtained under control conditions and after filament occlusion of the MCA origin (2h occlusion followed by 22h of reperfusion). Indicated are the ΔC_T_ values using elongation factor (EF)-1 as house-keeping gene as well as the fold up-regulation based upon the ΔΔC_T_ methodology. Given are mean ± SEM (≥ 4 samples) while the average fold change is indicated along with the 95% confidence intervals in brackets.

	Control	24 h after transient MCA occlusion			
		MCA ipsilateral		MCA contralateral	
	ΔC_T_	ΔC_T_	fold upregulation	ΔC_T_	fold upregulation
B_1_ mRNA	n.d.	0.88±0.42	*de novo expression*	1.30±0.39	*de novo expression*
B_2_ mRNA	2.7±0.4	-2.44±0.59**	x36 (16–81)	0.56±1.21*	x9.8 (2–51)

n.d., not detectable.

*p<0.05 vs. control

**p<0.01 vs. control MCA.

### Marked relaxation by BK occurring after transient MCA occlusion goes along with an upregulation of B_1_ and B_2_ receptor gene expression

**Fig 1:**
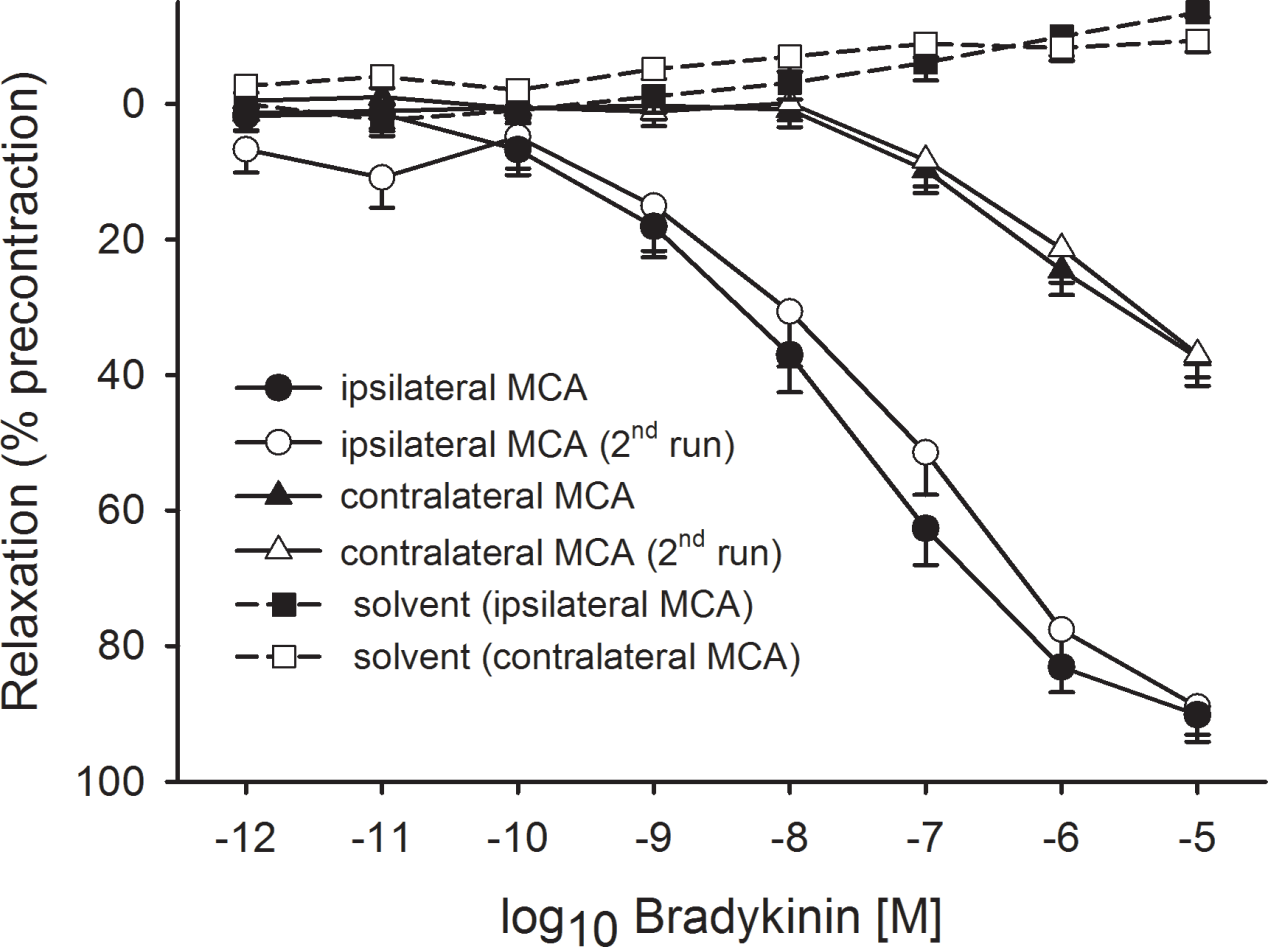
Concentration-related relaxation induced by bradykinin (BK) of middle cerebral artery (MCA) ring segments precontracted with U46619 (3x10^-7^ M). The ring segments were obtained from rats which had undergone a 2h MCA occlusion followed by 22h of reperfusion. Shown are the results for the right (ischemic) and left (contralateral) MCA. The vasomotor effects were comparable upon the first and second application of BK in both sides. Solvent indicates time matched solvent control measurements to check for the stability of precontraction. Given are mean ±SEM based on ≥7 observations.

After 2h of focal ischemia / 22h of recirculation BK induced a pronounced concentration-related relaxation which was well sustained in all MCA ring segments studied. This was true in the previously occluded MCA, and it was also true in the contralateral arteries, although at a significantly lower degree (Fig 1). A stable level of precontraction induced by U46619 (3x10^-7^ M) was found in time-matched solvent control experiments indicating that the relaxation was not due to spontaneous loss of tension. Furthermore, relaxation was fully reproducible when BK was applied repeatedly (Fig 1). Relaxation upon BK also occurred in segments precontracted by immersion in 50 mM K^+^ Krebs solution, although the maximum value observed (46±8.5%, n = 8) was considerably smaller than in U46619-precontracted segments.

The relaxation-inducing effect of BK went along with a significant upregulation of the B_2_ mRNA expression which was more pronounced in the MCAs from the ischemic hemisphere than in the contralateral arteries. In addition, there was a *de novo* expression of the B_1_ mRNA in the MCA vessel wall, again somewhat higher in the arteries taken from the ischemic than from the contralateral hemisphere (Table 1). These changes in gene expression were highly robust since they were found in all vessels studied.

### BK-induced relaxation after transient MCA occlusion is mediated by B_2_ receptor activation: functional and immunofluorescence evidence

**Fig 2:**
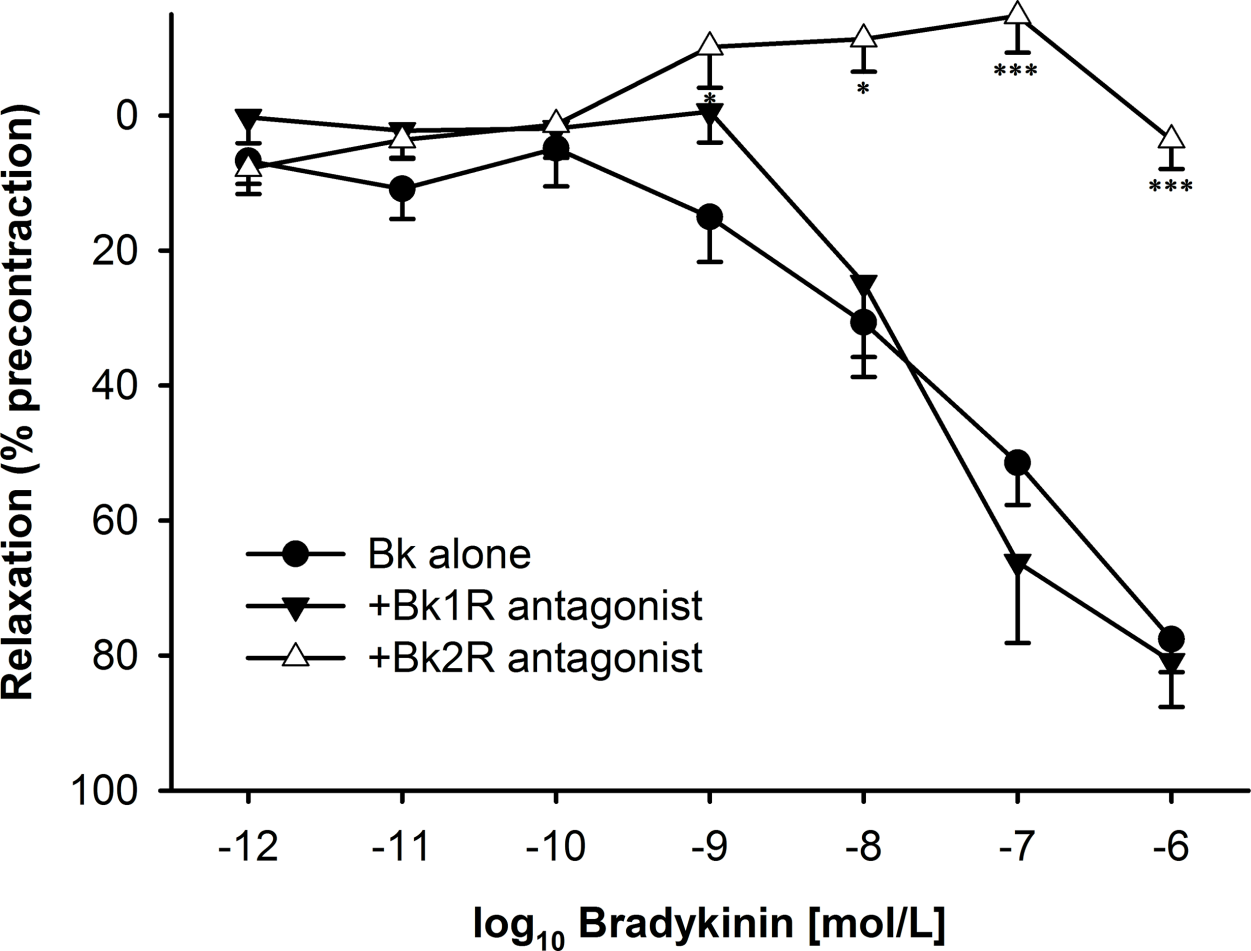
Effects of selective bradykinin (BK) receptor antagonists on the relaxation induced by bradykinin in rat middle cerebral artery (MCA) ring segments precontracted with U46619 (3x10^-7^ M). Segments were obtained 24h following transient MCA occlusion from the ischemic side. The BK-induced relaxation was not at all affected by the selective B_1_ receptor antagonist lys-(des-arg^9^, leu^8^)-BK (10^−5^ M) while it was completely abrogated in the presence of the selective B_2_ receptor antagonist Hoe140 (10^−6^ M). Indicated are mean ± SEM based on ≥6 observations. *p<0.05, **p<0.01 vs. BK alone.

In order to characterize the receptor subtype(s) underlying BK-induced relaxation following I/R injury, specific agonists and antagonist were used. In the ipsilateral MCA, the B_1_ agonist lys-(des-arg^9^)-BK up to 1μM was devoid of any vasomotor effect, both under resting tension conditions as well as in precontracted segments (not shown). Furthermore, BK-induced relaxation was completely unaffected in the presence of the B_1_ antagonist lys-(des-arg^9^,leu^8^)-Bk (10^−5^ M). In contrast, the selective B_2_ antagonist Hoe140 (10^−6^ M) abrogated the vasomotor effect of BK suggesting B_2_ activation to solely account for the relaxation induced by BK following I/R injury (Fig 2). Comparable effects were observed in ring segments from the contralateral MCA (not shown).

**Fig 3:**
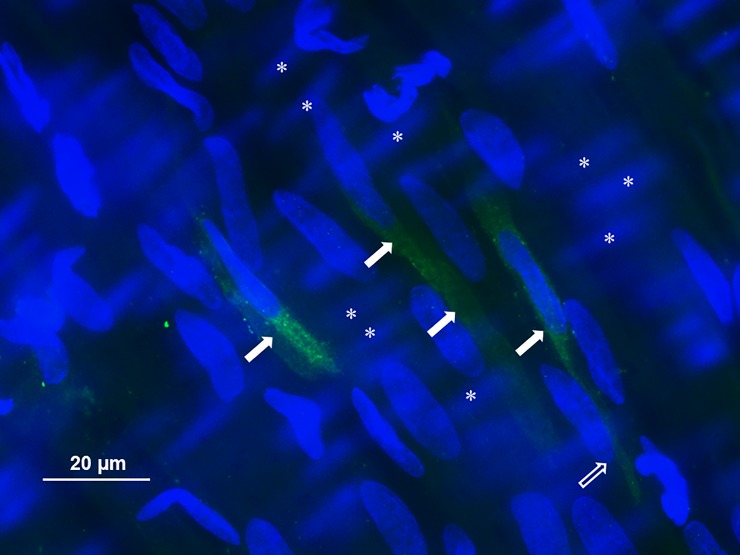
Whole mount immunofluorescence staining for the expression of B_2_ receptors in the middle cerebral artery wall following ischemia/reperfusion injury. Structures in blue are nuclei, while the B_2_ immunofluorescence presents in green. The endothelial cell nuclei run in the length axis of the artery while the smooth muscle cell nuclei are arranged perpendicular to the endothelial cells. Filled arrows mark endothelial cells displaying high level of immunofluorescence while the empty arrow points to an endothelial cell with low level staining. Asterisks mark smooth muscle cell nuclei (randomized choice) which are not in the focus of the image due to the thickness of the vessel wall. The clustering of endothelial cells displaying a high level of B_2_ receptor immunofluorescence was a typical finding, and we never found any immunofluorescent signal in smooth msucle cells.

Whole mount immunofluorescence staining to detect B_2_ receptor protein revealed cells displaying high fluorescent intensity either scattered or arranged in a cluster-like fashion in displaying a low or even undetectable intensity level. This is exemplified in an image shown in Fig 3. There was no signal detectable in smooth muscle cells in any artery studied. Control experiments with omission of the primary antibody did not show staining of the vessel wall. Furthermore, we never observed any significant immunofluorescence in MCAs taken from non-occluded animals.

### BK-induced relaxation after transient MCA occlusion is mediated by release of NO and activation of an EDHF-related pathway

**Fig 4:**
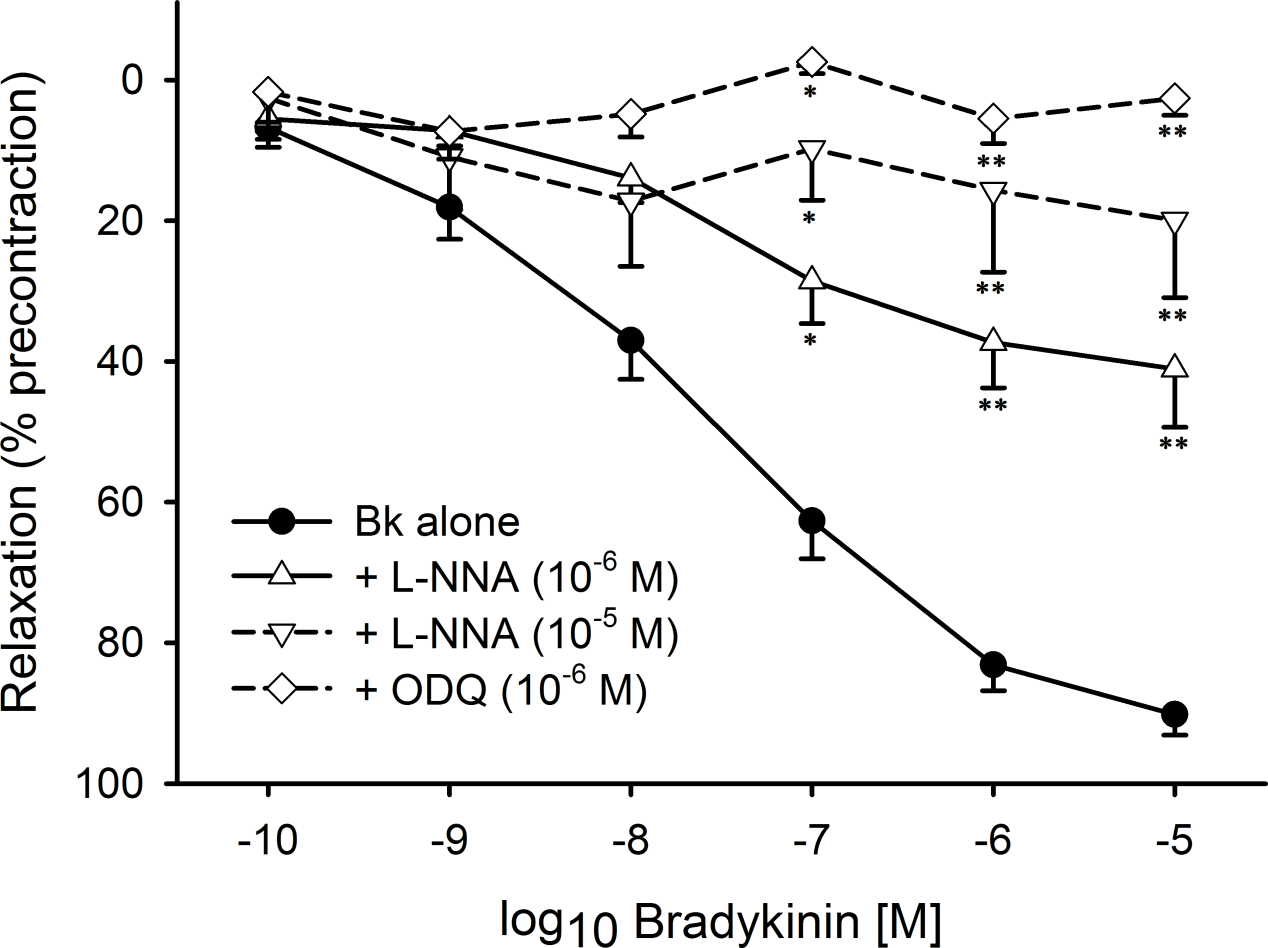
Role of the NO-cGMP axis in bradykinin-induced relaxation in rat middle cerebral arteries obtained 24h after induction of focal brain ischemia. Preincubation with N^ω^-nitro-l-arginine (L-NNA) inhibited BK-induced relaxation in a concentration-related manner. Similarly, inhibition of the soluble guanylyl cyclase by ODQ abrogated the effect of BK. Indicated are mean±SEM based on ≥ 4 observations.*p<0.05 vs control, **p<0.01 vs control.

We studied the role of NO in mediating BK-induced relaxation after transient MCA occlusion using 7-NI (10^−6^ M), a selective neuronal NOS (nNOS) inhibitor, 1400W (a selective inhibitor of the inducible NOS (iNOS), 10^−6^ M), and N^ω^–nitro-l-arginine (L-NNA, 10^−6^ M—10^−5^ M, an unselective NOS inhibitor). While 7-NI and 1400W did not significantly affect the BK-induced response, L-NNA inhibited relaxation in a concentration-related manner as shown in Fig 4. Furthermore, ODQ, a selective inhibitor of the soluble guanylyl cyclase completely blocked BK-induced relaxation. Moreover, L-NNA at 10^−5^ M and ODQ (10^−6^ M) both increased the basal contractile force of the segments indicating involvement of the NO-cGMP pathway in adjusting resting tension. These results suggest that NO released from the endothelial cells is of major functional importance in the control of wall tension and mediation of BK-induced relaxation. In accord, we found this response abrogated in endothelium-denuded segments (results not shown).

**Fig 5:**
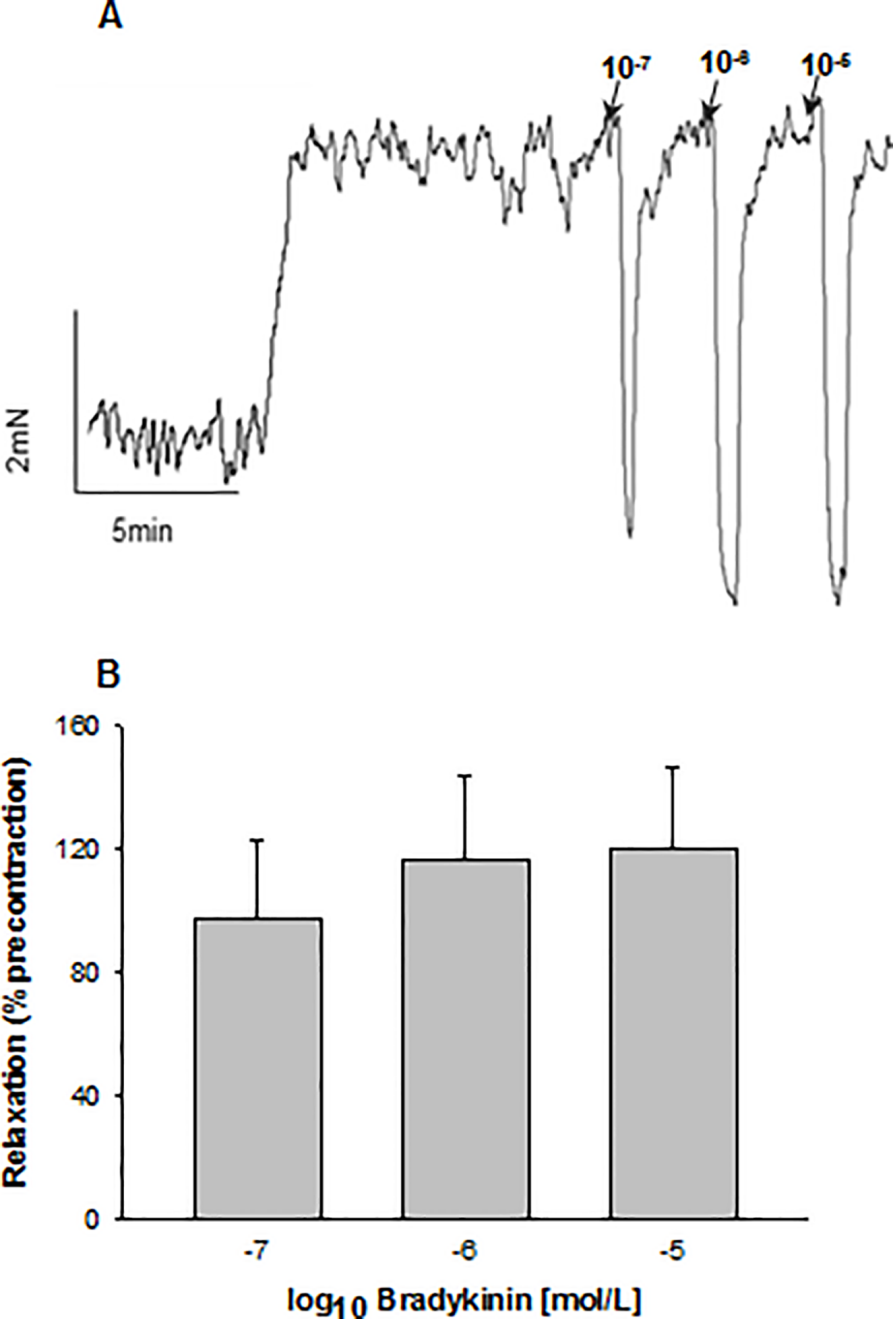
In the presence of N^ω^–nitro-l-arginine (L-NNA, 10^−5^ M) bradykinin (BK) elicits a transient relaxation in middle cerebral artery segments after transient focal brain ischemia. (**A**) Representative trace from a segment which was precontracted with U46619 (3x10^-7^ M). Application of high concentrations of BK (10^−7^ M to 10^−5^ M) induced transient peaks of relaxation. (**B**) Mean values of the transient peaks of relaxation induced by high concentrations of BK in segments obtained from the previously occluded middle cerebral artery. Indicated are mean±SEM (n = 5).

Although NO released from endothelial cells was identified to play a major role in mediating BK-induced relaxation following transient MCA occlusion, there is evidence in favor of a membrane hyperpolarization-induced mechanism to be involved as well. This evidence is based on several observations including (i) a marked decrease of the maximal relaxation achieved with BK following precontraction in a 50 mM K^+^ Krebs solution as mentioned above, and (ii) the occurrence of a transient relaxant response in the presence of 10^−5^ M L-NNA (but not with 10^−6^ M) as shown in Fig 5 and similarly in the presence of ODQ (10^−6^ M) (not shown).

**Fig 6:**
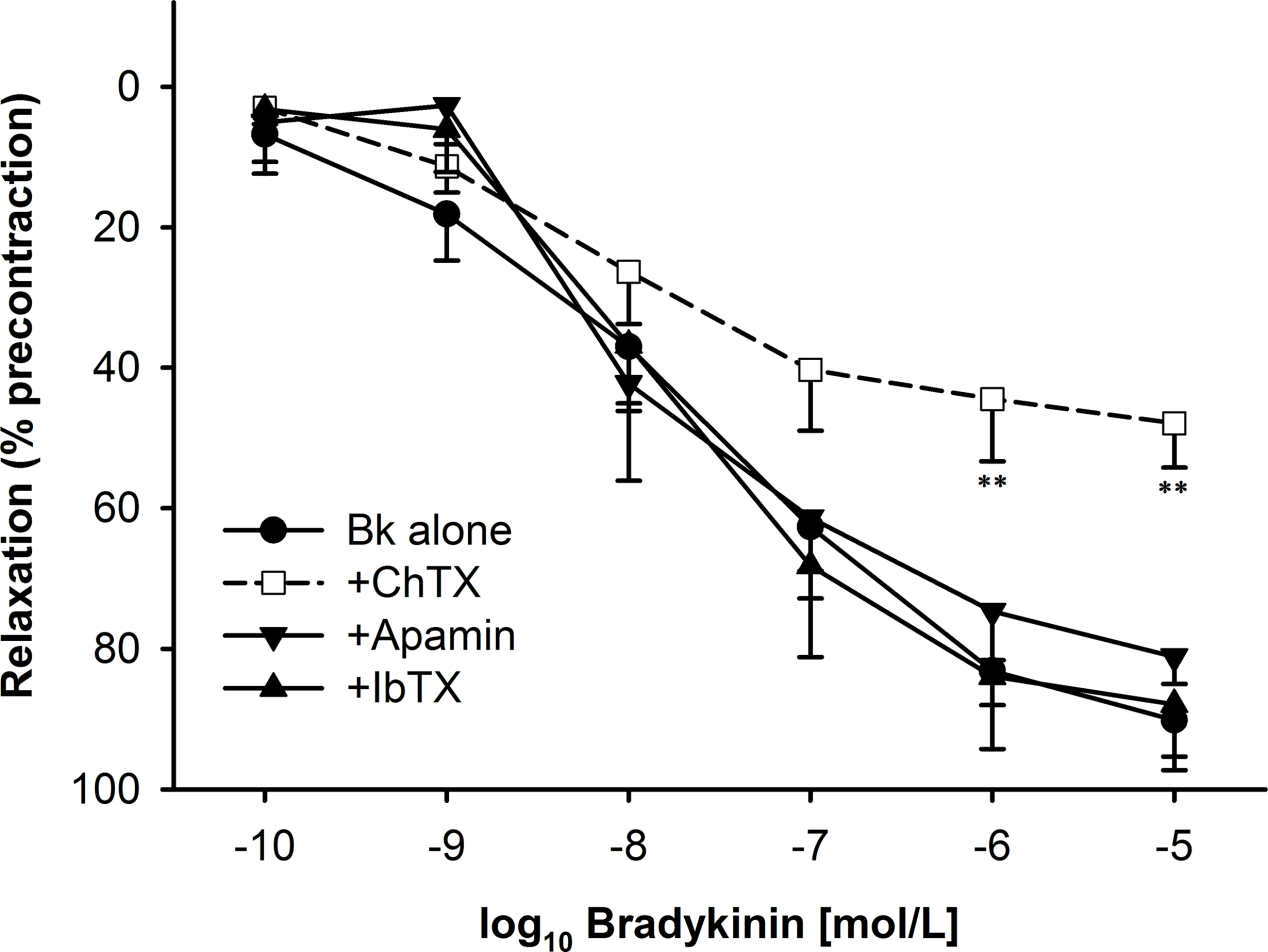
Effects of potassium channel blockers on the relaxation induced by bradykinin (BK) in middle cerebral artery segments after ischemia/reperfusion injury. The response to BK was partially inhibited by charybdotoxin (ChTx, 10^−8^ M, n = 8), but did not change in the presence of apamin (10^-7^M, n = 7) or iberiotoxin (IbTx, 10^−8^ M, n = 6). Indicated are mean±SEM. ** p<0.01 vs. control.

In order to elucidate the mechanism involved in the generation of the transient relaxant response, we preincubated segments with different inhibitors of K_Ca_ channels before precontraction with U46619 and administration of BK. The results shown in Fig 6 indicate that ChTx decreased the relaxant response to high concentrations of BK while neither apamin nor iberiotoxin significantly affected BK-induced relaxation. In addition, ChTx increased the inhibitory effect of L-NNA (10^−6^ and 10^−5^ M each) on the sustained phase of the BK-induced response, but this effect did not reach statistical significance. Moreover, the transient relaxation upon BK occurring in MCA segments with the NO-cGMP axis inhibited (see Fig 5) was completely suppressed by ChTx. Based on the spectrum of actions with ChTx blocking large and intermediate conductance channels and iberiotoxin and apamin acting as selective blockers of large and small conductance channels [26] these results strongly suggest the involvement of intermediate conductance K_Ca_ channels underlying the EDHF-related pathway activated by BK.

## Discussion

The present study describes a significantly enhanced relaxant action of BK in the isolated rat MCA following transient focal brain ischemia. Enhanced relaxation was observed in MCAs from the ischemic as well as from the contralateral hemisphere with the response being more pronounced ipsilaterally. Relaxation was accompanied by a significant upregulation of the B_2_ and a *de novo* expression of the B_1_ mRNA in the MCA wall, again more pronounced ipsi- than contralaterally. Whole mount immunofluorescence staining revealed the presence of B_2_ receptor protein in previously occluded MCAs but not in arteries taken from non-occluded rats. Relaxation was mediated by B_2_ activation exclusively as shown in experiments using selective receptor antagonists. The relaxation was endothelium-dependent with release of NO playing a major role. In addition, an EDHF-related pathway, apparently involving intermediate conductance K_Ca_ channel activation appeared to be involved as well.

In the majority of reports published, BK induced relaxation of isolated cerebral arteries from different species including man [27–37]. However, there are also reports available on a low efficacy or even a lack of vasodilator action of BK in rat MCAs [38, 39]. Thus, our results in segments obtained from control rats are not at all unique. The reason for this unexpected result in control arteries is not clear, however, endothelial damage can be excluded based on the marked relaxation induced in all segments by the selective endothelin B receptor agonist sarafotoxin 6c, well in line with previous observations [40]. Recent studies in our lab revealed a moderate although significant relaxation by BK in MCAs from Wistar rats, well in accord with data from the literature [32, 41, 42]. Thus, the lack of effect in the Sprague-Dawley rat MCAs used in the present study is most probably a strain-specific feature. Interestingly, the B_2_ receptor mRNA was detectable in all MCAs from control rats studied, although we could not demonstrate the presence of B_2_ receptor protein using immunofluorescence staining. Thus, a low level of protein expression appears to be the most feasible explanation for the lack of BK-induced vasoactive responses in MCA segments from control rats.

In contrast to the results obtained in control arteries, BK induced marked relaxation 24h after induction of I/R injury. This robust relaxation went along with a significant upregulation of the B_2_ mRNA and a *de novo* expression of the B_1_ mRNA in the vessel wall. In this context, one has to bear in mind that the MCA is not directly affected by the filament which in fact is positioned within the internal carotid artery so as to occlude the MCA origin. Therefore, direct mechanical disturbance/stimulation of the MCA wall as a trigger for the changes in our functional and gene expression studies can well be excluded. Upregulation of the B_2_ and *de novo* expression of the B_1_ receptor may be considered part of an inflammatory response, all the more as further components of the KKS, most notably kininogen expression was also found upregulated in the MCA following induction of I/R injury (own results). These observations strongly suggest the presence of a complete KKS in the vascular wall implying independence from feeding with BK or its precursor from the blood as suggested previously in a model of focal brain injury [43]. The assumption of a vascular inflammatory response may also hold true for the alterations seen in the contralateral MCA, although at a somewhat lower level. These alterations may contribute to the phenomenon termed crossed hemodynamic diaschisis, a decrease in blood flow in the contralateral cerebral and/or cerebellar hemisphere as repeatedly described in patients suffering from a unilateral stroke event [44, 45] and in animal experiments on focal brain ischemia [46–48]. Pathophysiologically, hemodynamic diaschisis is considered a consequence of diminished cerebral metabolism due to disturbances of neuronal connections between the site of damage and remote regions assuming neurovascular coupling to be intact. However, regions of hypometabolism and hypoperfusion may not necessarily coincide [49]. Our present results suggest that endogenous reaction pathways in the vessel wall including changes in gene expression may significantly affect control of cerebrovascular tone and reactivity even in remote vessel sections under pathological conditions. Thus, crossed hemodynamic diaschisis appears to be more complex than hitherto thought.

In principle, both BK receptor subtypes may be involved in the control of vasomotor tone and blood pressure regulation [50]. However, use of selective agonists and antagonists showed that only B_2_ receptor activation accounted for the relaxation-inducing effect of BK in the present study. Thus, the functional role of B_1_ receptor expression in the vessel wall following I/R injury remains obscure. The relaxation due to B_2_ receptor activation mainly involves the NO-cGMP axis since it could be blocked by L-NNA and ODQ. In addition to eNOS, we also considered nNOS and iNOS as potential sources of NO release based on previous reports [51, 52]. However, neither 7-NI nor 1400W, selective inhibitors of nNOS and iNOS activity significantly affected BK-induced relaxation suggesting eNOS to be the only functionally important source of NO mediating BK-induced relaxation in our vessel segments. In preliminary studies we did not find evidence in favor of an upregulation of the eNOS message in the vessel wall, rather, the eNOS mRNA level appeared to be somewhat downregulated. Therefore, the *de novo* relaxation induced by BK following induction of I/R injury appears to be due to an increased B_2_ receptor expression as shown here on the level of gene expression. An increase in receptor density will augment Ca^2+^ ion entry into endothelial cells upon binding of BK resulting in a stronger stimulation of the Ca^2+^-dependent eNOS activity. Moreover, the higher level of intracellular Ca^2+^ concentration apparently activated a second relaxation-eliciting pathway, apparently mediated by membrane hyperpolarization. Such a pathway has been described to be present in many vascular beds with experimental evidence in favor of a variety of putative factors and mechanisms depending on the species and vascular bed as reviewed recently [53, 54]. However, in many blood vessels, the increase of the intracellular Ca^2+^ ion concentration following activation of a G-protein coupled receptor such as the B_2_ leads to activation of K_Ca_ channels with K^+^ ion efflux and membrane hyperpolarization. Involvement of an EDHF-related pathway in the present study is suggested by (i) a significantly decreased BK-induced relaxation (approximately 50%) following precontraction with a depolarizing Krebs solution (containing 50 mM K^+^) and (ii) a significantly decreased BK-induced relaxation (again approximately 50%) following preincubation with the K_Ca_ channel blocker ChTx. The latter result in conjunction with the observation that neither apamin nor iberiotoxin significantly affected BK-induced relaxation suggests that K_Ca_ channels of intermediate conductance were activated. The presence of this K_Ca_ channel subtype in endothelial cells is well established [55]. Spread of the endothelial hyperpolarization onto the smooth muscle cells may occur via myo-endothelial gap junctions which have been found in rat cerebral arteries [56] or the accumulation of K^+^ ions in the interstitial space within the vessel wall with subsequent activation of the Na/K-ATPase and inward rectifying K^+^ channels in the smooth muscle cells [57, 58].

The occurrence of a functionally important EDHF-related pathway in the MCA following ischemia-reperfusion injury is in line with previous studies [53, 59]. Activation of this pathway became apparent only when the NO-cGMP axis was inhibited and subsequently BK-induced relaxation abrogated. Thus, an indomethacin-sensitive contribution was virtually not involved which is in accord with previous results obtained in cerebral arteries from Sprague-Dawley rats [60]. The appearance of an EDHF-mediated relaxation with high concentrations of BK may be explained by data indicating that such a pathway requires a higher intraendothelial Ca^2+^ concentration than eNOS stimulation [61]. In the absence of L-NNA activation of eNOS may well have superimposed and masked the activation of the EDHF-related pathway.

In conclusion, the present study adds BK to a couple of agonists which are characterized by altered vasomotor effects in cerebral arteries under pathological conditions such as focal ischemia. While these compounds including endothelin 1, angiotensin II, and serotonin have been described to act upon the smooth muscle cells, the present study is the first to provide evidence in favor of an altered endothelial cell function following I/R injury. The main effector appears to be a significant upregulation of the underlying B_2_ receptor expression.
